# Nrf2 activation rescues stress-induced depression-like behaviour and inflammatory responses in male but not female rats

**DOI:** 10.1186/s13293-024-00589-0

**Published:** 2024-02-13

**Authors:** Ryan T. McCallum, Rachel-Karson Thériault, Joshua D. Manduca, Isaac S. B. Russell, Angel M. Culmer, Janan Shoja Doost, Tami A. Martino, Melissa L. Perreault

**Affiliations:** Department of Biomedical Sciences, University of Guelph, 50 Stone Rd. E., Guelph, ON N1G 2W1 Canada

**Keywords:** Sex differences, Major depressive disorder, Neuroinflammation, Nuclear factor erythroid 2-related factor 2, Dimethyl fumarate

## Abstract

**Background:**

Major depressive disorder (MDD) is a recurring affective disorder that is two times more prevalent in females than males. Evidence supports immune system dysfunction as a major contributing factor to MDD, notably in a sexually dimorphic manner. Nuclear factor erythroid 2-related factor 2 (Nrf2), a regulator of antioxidant signalling during inflammation, is dysregulated in many chronic inflammatory disorders; however, its role in depression and the associated sex differences have yet to be explored. Here, we investigated the sex-specific antidepressant and cognitive effects of the potent Nrf2 activator dimethyl fumarate (DMF), as well as the associated gene expression profiles.

**Methods:**

Male and female rats were treated with vehicle or DMF (25 mg/kg) whilst subjected to 8 weeks of chronic unpredictable stress. The effect of DMF treatment on stress-induced depression- and anxiety-like behaviours, as well as deficits in recognition and spatial learning and memory were then assessed. Sex differences in hippocampal (HIP) gene expression responses were also evaluated.

**Results:**

DMF treatment during stress exposure had antidepressant effects in male but not female rats, with no anxiolytic effects in either sex. Recognition learning and memory and spatial learning and memory were impaired in chronically stressed males and females, respectively, and DMF treatment rescued these deficits. Further, chronic stress elicited sex-specific alterations in HIP gene expression, many of which were normalized in animals treated with DMF. Of note, most of the differentially expressed genes in males normalized by DMF were related to antioxidant, inflammatory or immune responses.

**Conclusions:**

Collectively, these findings may support a greater role of immune processes in males than females in a rodent model of depression. This suggests that pharmacotherapies that target Nrf2 have the potential to be an effective sex-specific treatment for depression.

## Abstract (plain language summary)

Major depressive disorder is two times more prevalent in females than males. Further, immune system dysfunction has been shown to contribute to the development of depression, with previous studies consistently reporting chronic low-grade inflammation in depressed individuals. Not surprisingly, the immune system dysfunction associated with depression appears to be sex specific. As such, whilst anti-inflammatory drugs have shown antidepressant effects in preclinical studies, the sex differences in these effects are seldomly investigated. Thus, this study sought to determine the sex-specific antidepressant and cognitive effects of dimethyl fumarate (DMF) treatment. DMF is a drug that activates the protein nuclear factor erythroid 2-related factor 2 to initiate anti-inflammatory processes. Here, male and female rats were exposed to 8 weeks of chronic stress whilst receiving daily DMF treatment. Subsequently, their expression of depression- and anxiety-like behaviours, as well as learning and memory deficits were assessed. Alterations in gene expression were also evaluated. DMF treatment had antidepressant effects in male rats only but did not have anti-anxiety effects in either sex. The learning and memory deficits in both sexes were rescued with DMF treatment. Notably, DMF normalized several of the sex-specific gene alterations induced by chronic stress, with many of the male-specific genes relating to inflammatory processes. These data suggest that DMF may be an effective antidepressant treatment in males.

## Highlights

- Dimethyl fumarate had antidepressant effects in chronically stressed male rats only.
- Dimethyl fumarate rescued sex-specific learning and memory deficits induced by chronic stress.
- Male and female rats exhibit different gene expression responses to chronic stress, with male rats exhibiting more alterations in inflammatory-associated genes than female rats.
- Dimethyl fumarate normalized the expression of several gene transcripts in stress-exposed male and female rats.

## Background

Major Depressive Disorder (MDD) is a recurrent multidimensional affective disorder with a lifetime prevalence ranging from 2 to 21% worldwide [1, 2]. Given that chronic stress is an established risk factor for MDD development [3] and that females consistently have a greater behavioural sensitivity to chronic stress exposure [4–7], it is not surprising that the prevalence of MDD is two times greater in women than in men [8]. Based on the traditional monoamine hypothesis of depression, the majority of antidepressant pharmacotherapies center on enhancing central nervous system (CNS) monoamine neurotransmission [9]. However, this hypothesis is now largely accepted as a simplification of depression pathophysiology; a concept supported by the high rates of treatment resistance and relapse associated with traditional antidepressant pharmacotherapy use [10, 11]. Rather, recent evidence supports multi-system dysfunction in MDD that warrants a multi-target approach for novel sex-specific therapeutic interventions [12–14].

Immune system dysfunction has emerged as a major contributing factor for depression [14]. Specifically, psychological stress has been reported to initiate proinflammatory cascades [15, 16]. Thus, it follows that chronic low-grade inflammation has become a distinct feature in both clinical presentations [17–21] and preclinical models of MDD [22–26]. Clinically, increased CNS microglial activation [20, 21] and plasma proinflammatory cell proliferation have been reported in MDD patients compared to healthy controls. Further, elevated proinflammatory cytokines are a robust phenotype of adult MDD [18, 19, 27, 28], with elevated serum levels of several cytokines correlating with depressive symptom severity and suicidality [19, 27, 29, 30]. In line with the clinical findings, increased microglial activation has been observed within the hippocampus (HIP) of mice in chronic despair [26] and chronic unpredictable stress (CUS) [25] models of depression, as well as in the prefrontal cortex (PFC) of rats exposed to chronic restraint stress [31]. In addition, levels of proinflammatory Th17 cells were increased in the mouse HIP following CUS [25] and in the whole brain of mice following chronic restraint test and learned helplessness [24]. CUS exposure has also been demonstrated to elicit increased mRNA expression of several proinflammatory cytokines in the HIP of male mice [22, 25] and rats [23]. Notably, the sex differences observed in depression appear to extend to the associated immune system dysfunction. For instance, at baseline female rats reportedly had greater microglial activation in the PFC than male rats; this effect was reversed following acute and chronic restraint stress [32]. Moreover, whilst Liu et al. [33] found increased microglial activation in the HIP of both male and female mice following CUS, the stress-induced alterations in cytokine expression were sex-specific. As such, findings to date highlight the importance of including sex as a variable when investigating the neuro-immune axis in the context of MDD.

The transcription factor nuclear factor erythroid 2-related factor 2 (Nrf2) is a key regulator of antioxidant signalling during inflammation [34]. Following oxidative stress, Nrf2 is stabilized and induces the expression of antioxidant and cytoprotective genes, including *heme oxygenase-1 (HO-1), NAD(P)H quinone dehydrogenase 1 (NQO1)* and *glutamate cysteine ligase (GCL)* [34–36]. Thus, Nrf2 dysregulation commonly accompanies disorders associated with chronic inflammation [34], but it has yet to be explored in MDD. Preclinical investigations, however, that have used animal models to study aspects of depression have effectively demonstrated the impact of chronic stress exposure on Nrf2 expression. Male rats that exhibited CUS-induced depression-like behaviours also had decreased *Nrf2, HO-1* and *NQO1* mRNA and Nrf2 protein levels in the HIP [37]. Further, subsequent treatment of these rats with curcumin, an Nrf2 activator, reversed these behavioural and molecular phenotypes [37]. Similarly, in both the social defeat stress (SDS) and lipopolysaccharide (LPS) models of depression, male mice showed reduced Nrf2 protein levels in the PFC and HIP; a result that correlated with depression-like behaviours and that was reversed following Nrf2 activation with sulforaphane [38, 39]. Of note, Nrf2 knockout (KO) mice display a depressive phenotype [38, 40], increased serum cytokine levels, as well as increased microglial activity in the PFC and HIP [40].

Based on these findings, the potent Nrf2 activator DMF is currently under investigation for its potential as an antidepressant [23, 41, 42]. Previous studies have illustrated the ability of DMF treatment to mitigate CUS-induced behavioural despair and anhedonia in male mice [41, 42] and rats [23]. Moreover, in these same studies, DMF treatment improved recognition learning memory in the novel object recognition test [42], reversed stress-induced HIP microglial activation [42], reduced mRNA [23] and protein [42] levels of the proinflammatory cytokines interleukin (IL)-1β and tumour necrosis factor (TNF)-α in the HIP, as well as decreased HIP levels of the oxidative stress marker malondialdehyde [23]. Taken together, these results support DMF as a potent anti-inflammatory with promising antidepressant effects in male subjects. However, whilst there is evidence to suggest sexually dimorphic effects of DMF treatment in rodent models of experimental autoimmune encephalomyelitis (EAE) [43] and Alzheimer’s disease (AD) [44, 45], its sex-specific antidepressant effects have yet to be elucidated.

Therefore, to better understand the sex-specific antidepressant and cognitive effects of DMF, we evaluated the effect of chronic DMF treatment on depression- and anxiety-like behaviours, learning and memory, and HIP gene expression profiles in both male and female rats exposed to CUS.

## Methods

### Animals

Sixty male and female young adult Wistar rats of approximately the same age were used, with females weighing on average 210 g and males weighing on average 250 g at the beginning of the study. Following arrival, rodents were housed in same sex groups of three in ventilated cages with access to water and food ad libitum. Rodents were housed in a temperature-controlled colony room (temperature 21 °C, humidity 30–45%) and maintained on a 12-h reverse light–dark cycle (08:00 lights off; 20:00 lights on) unless otherwise noted. On the first day of the CUS protocol rodents were relocated to non-ventilated, wire-roofed, opaque individual cages for the remainder of the study. No enrichment was provided, and animals were food restricted to 15–20 g of 18% rodent chow daily, representing approximately 80% of their standard daily intake. All behavioural experiments were conducted in a red light illuminated room during the dark phase of the day–night cycle when rodents were active. All experimental procedures and protocols were approved by the Animal Care Committee of the University of Guelph and carried out in accordance with the recommendations of the Canadian Council on Animal Care.

### Drugs

DMF was purchased from Sigma-Aldrich (St. Louis, MO) and administered orally at a dose of 25 mg/kg [23]. Rodents were habituated to daily consumption of 1 g of peanut butter for 5 days with DMF uniformly mixed in once CUS began. Untreated peanut butter was used as the vehicle (VEH) condition. All rats consumed 100% of the peanut butter within 5–10 min. Stainless steel feeding dishes with DMF-infused and VEH peanut butter were placed into rodent cages daily at 08:00 h and removed at 08:15 h. A 15-min feeding period allowed for consumption of the full dose.

### Chronic unpredictable stress

The CUS animal model of depression is the most widely used rat model system for the study of depression as it has the greatest validity and translational potential [46–48]. The CUS schedule used in this investigation was a modified version of the protocol described previously [49]. Developed to last a total of 8 weeks, the current protocol consisted of various uncontrollable, non-debilitating, and inescapable physical, psychological, and circadian stressors [49]. Stressors varied daily and included: cage tilt (30–45^0^, 8 h), cold swim (10–13 ℃, 5 min), cold exposure (4 ℃, 1 h), damp bedding (500 mL water, 12–14 h), food and water deprivation (24 h), lights on and off intermittently (8 h), and reverse light cycle (24 h). Each stressor was only administered once in any given week, with each weekly schedule generated randomly to prevent predictability. Stressors were continued throughout the behavioural testing period to maintain the depression-like phenotype.

### Experimental design

Rodents were randomly assigned to one of six experimental groups (*N* = 10 subjects per group) and included: male VEH-treated non-stressed control (male VEH); male VEH-treated CUS (CUS-VEH); male DMF-treated CUS (CUS-DMF); female VEH-treated non-stressed control (female VEH); female CUS-VEH; female CUS-DMF. Rodent weights were collected twice per week to monitor rodent health and adjust DMF dosage accordingly.

### Forced swim test

The forced swim test was used to evaluate behavioural despair and any antidepressant effects of DMF [49, 50]. Rodents were placed into a 50-cm-tall Plexiglas cylinder filled with 24 ± 1 °C water to the height of 35 cm on 2 consecutive days. On day 1, rodents were placed into the cylinder for 15 min for the pre-test. 24 h later, rodents were placed in the cylinder once again for 5 min. Animals were recorded and at 5-s intervals the following parameters were measured: climbing (both front paws breaking the surface of the water whilst attempting to jump out of the cylinder), swimming (movement of limbs paddling across the water surface) or immobility (passive floating with movements only necessary to keep nose above water).

### Sucrose preference test

The sucrose preference test was used to investigate anhedonia and pleasure-seeking behaviour [49]. Prior to the first test, two bottles of 1% (w/v) sucrose solution were placed in each cage for 24 h for the animals to habituate to the sucrose solution. For the following 24 h, one bottle of sucrose solution was replaced with water. Once habituation was complete, animals were deprived of food and water for 24 h, after which the sucrose preference test was conducted. Animals were given two pre-weighed bottles: one containing 1% sucrose solution and one containing water. Bottles were counterbalanced between cages and switched after every measurement. The bottles were re-weighed every hour for 3 h. The percent sucrose preference (volume sucrose solution consumed/total volume consumed * 100) was calculated.

### Novel object recognition

The novel object recognition test was used to measure recognition learning and memory [51, 52]. Rodents were habituated to an open field measuring 100 cm × 100 cm × 40 cm. Habituation lasted 2 consecutive days with a single 10-min exposure on day 1 and two 10-min exposures on day 2. Testing took place on the third day. Rodents were placed into the open field facing a corner. Animals were allowed to explore two identical objects in each corner of the arena over a 4-min acquisition phase followed by a 3-min test phase 2 h later. During the test phase one of the objects was switched for a novel, unfamiliar object. The novel object was counterbalanced between rat trials with both the objects and arena cleaned between animals. The time spent exploring each object was recorded. Exploration was strictly defined as sniffing, touching, and facing the object in proximity. The discrimination ratio was calculated as the time exploring the novel object - the time exploring the familiar object / total exploration time.

### Object location

The object location test was used to measure spatial learning and memory [51, 52]. The OL test was comprised of an acquisition phase and test phase, both 3 min in length respectively and separated by a 5-min delay. The acquisition phase consisted of two identical objects placed in the adjacent corners of the arena and in the test phase one object was moved to the opposite corner. Objects were counterbalanced and the area cleaned between phases and each animal. The discrimination ratio was calculated as the time exploring the novel location - the time exploring the familiar location / total exploration time.

### Novelty suppressed feeding task

The novelty suppressed feeding task was employed to assess anxiety-like behaviour [53, 54] and utilizes the feeding motivation of food deprived rodents to measure exploration in an illuminated novel environment. At 48 h before testing, rodents were deprived of all food pellets for 24 h. Following this, rodents were given a brief 2-h window to feed followed by further deprivation until after the testing period on the following day. On the day of behavioural testing, the centre of the open field was illuminated (250 lumens) where a glass petri dish containing a single 18% rodent chow food pellet freshly submerged in 50% sucrose water was placed. The experimental parameters analyzed included latency to feed, and latency to first center approach with an approach was described as contact with the petri dish. A time cut-off of 10 min was established before conducting this experiment. Immediately following the task, rats were returned to their home cage, transferred to an isolated room, and provided a single food pellet weighing ~ 5 g. For 10 min, rodents were monitored and scored by an experimental examiner for in-cage feeding latency and in-cage total food consumption. Concluding this in-cage observation, rodents were given 10–15 g of 18% rodent chow to compensate for the food deprivation and returned to the colony cage racks.

### Gene expression

Following the behavioural tests, rats underwent rapid decapitation and dorsal HIP tissue was dissected. Total RNA was isolated from HIP tissue of male and female animals using the RNeasy Mini Kit (Qiagen, Hilden, Germany) (*N* = 4/group), as previously described [55]. RNA quantity and quality were assessed by NanoDrop ND-1000 (260/280 > 2; ThermoScientific) and by RNA ScreenTape (RIN ≥ 7; Agilent). Whole-genome microarray analyses were performed with the Affymetrix GeneChip Rat Gene 2.0 ST microarray, which interrogates > 27,000 protein coding transcripts, with a median of 22 probes per gene, and reproducibility at a signal correlation coefficient > 0.99. All microarray data are accessible (GEO Accession #GSE248186). Bioinformatics analyses were performed on GeneSpring GX 14.9.1 (Agilent Technologies Inc). Raw CEL files were uploaded into a project file with exon analysis and Affymetrix exon expression experiment settings and a biological significance workflow analysis. The rat gene 2.0 ST annotation technology (RaGene-2_0-st-na33_2_rn4_2013-03-28) was used for all analyses. Raw fluorescence data were normalized across all chips in each study, with a lower threshold of > 60 Raw Fluorescence Units (RFU) in at least 50% of conditions. Principal Components Analysis (PCA) was used for group level clustering. Gene ontology (GO) analysis for enriched genes was performed using the NIH DAVID Bioinformatics Database, Functional Annotation Tool.

### Statistical analysis

Normality tests and Levene’s test for equality of variance were employed for all data. Within sex treatment group differences were analyzed using a one-way analysis of variance (ANOVA) with Treatment as the between subjects’ variable and Tukey or Games-Howell post-hoc tests as appropriate. All values represented are expressed as group mean ± standard error (SEM). Statistical analysis was performed with IBM SPSS software (Version 24, Armonk, USA).

## Results

**Fig. 1:**
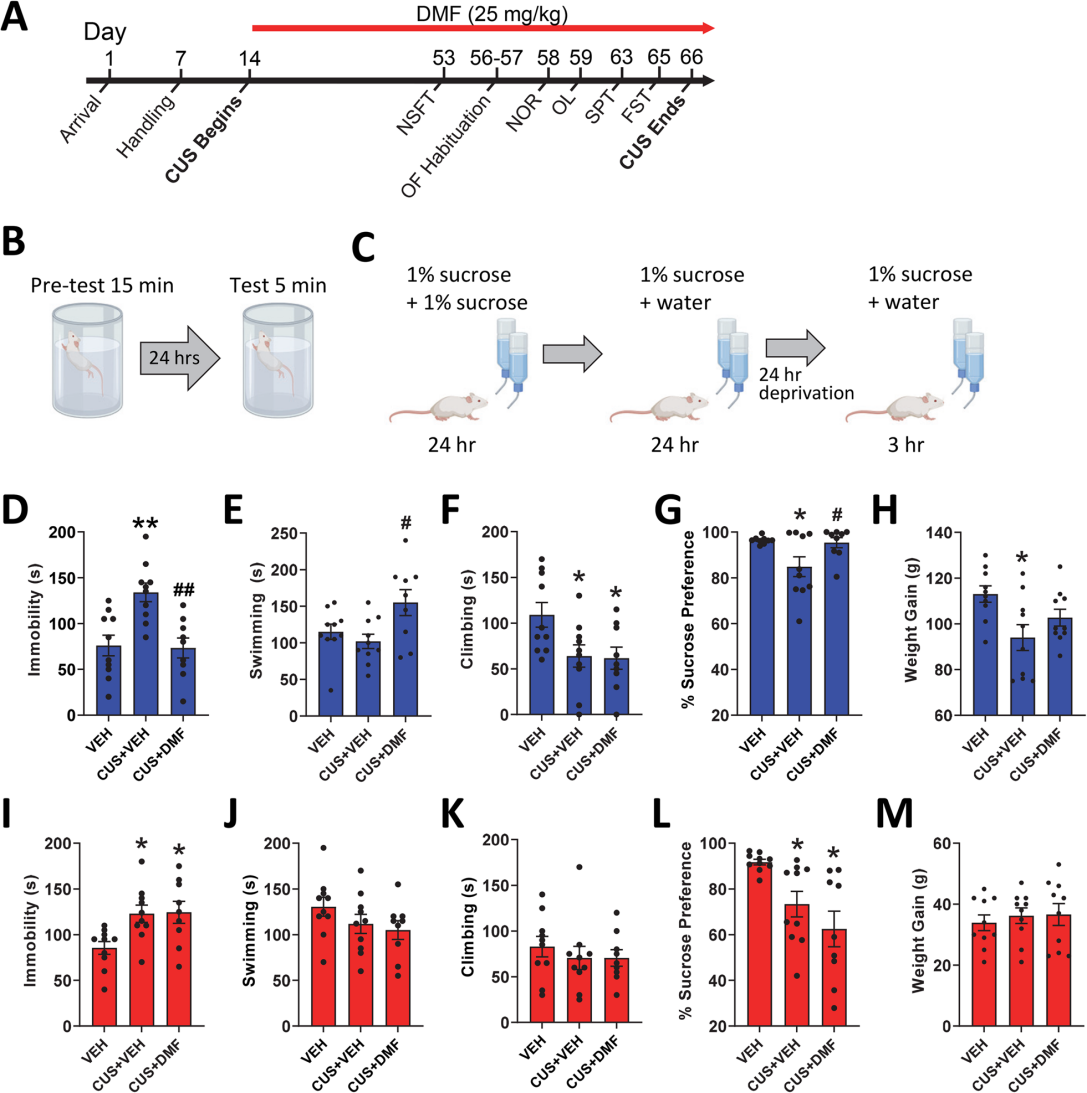
The effect of DMF treatment on CUS-induced depression-like behaviours and weight gain. **A** The full experimental timeline is shown. **B**, **C** Timelines for the forced swim test and sucrose preference test. Effect of CUS alone or combined with DMF on **D–F** immobility, swimming, and climbing behaviour in male rats during the forced swim test. **G** Male rats exposed to CUS displayed anhedonia-like behaviour in the sucrose preference test that was normalized by DMF **H** group differences in weight gain in the male rats. Effect of CUS alone or combined with DMF on **I-K** immobility, swimming, and climbing behaviour in female rats during the forced swim test. **L** CUS decreased sucrose preference with no effect of DMF. **M** Group differences in weight gain in the female animals. The data are presented as mean ± SEM of N = 9–10 animals per group. *p < 0.05 and **p < 0.01 compared to vehicle and #p < 0.05 and ##p < 0.01 compared to CUS + vehicle. A one-way ANOVA followed by a Tukey or Games-Howell post-hoc test was used for all analyses

The sex-specific effects of daily DMF administration on CUS-induced behaviours were evaluated with the overall experimental timeline depicted in Fig. 1A. Graphics showing the timeline of the forced swim test and sucrose preference test are depicted in Fig. 1B and C. In male rats, CUS elevated immobility time (*p* = 0.002) and reduced climbing behaviour (*p* = 0.01) in the forced swim test, with no effects on total time spent swimming (Figs. 1D–F). DMF treatment to CUS-exposed rats normalized immobility time but not climbing behaviour, and significantly elevated active swimming time (*p* = 0.02, Fig. 1E). Sucrose preference was suppressed by CUS in the male rats (*p* = 0.04, Fig. 1G), effects that were abolished by DMF treatment (forced swim test: immobility F(2,26) = 10.2, *p* < 0.001, swimming F(2, 26) = 4.5, *p* = 0.02, climbing F(2, 26) = 4.5, *p* = 0.02; sucrose preference test: F(2, 25) = 4.8, *p* = 0.02). Over the course of the study, CUS exposure resulted in lower weight gain in the male animals (*p* = 0.01, Fig. 1H), an effect that showed only minor mitigation by DMF such that the mean weights of DMF-treated rats fell in between controls and the vehicle-treated CUS-exposed group, with no statistical difference from either. In female rats, CUS elevated forced swim test immobility time (*p* = 0.02, Fig. 1I) with no effects on swimming or climbing (Fig. 1J, K). Unlike that observed in the males, DMF treatment did not ameliorate the effects of CUS on immobility or alter swimming or climbing behaviour in the female rats. Similarly, CUS lowered sucrose preference (*p* = 0.05, Fig. 1L) with no effects of DMF treatment [forced swim test: immobility F(2,26) = 5.5, *p* = 0.01; sucrose preference test: F(2, 26) = 7.5, *p* = 0.003]. CUS did not influence weight gain in the female animals (Fig. 1M).

**Fig. 2:**
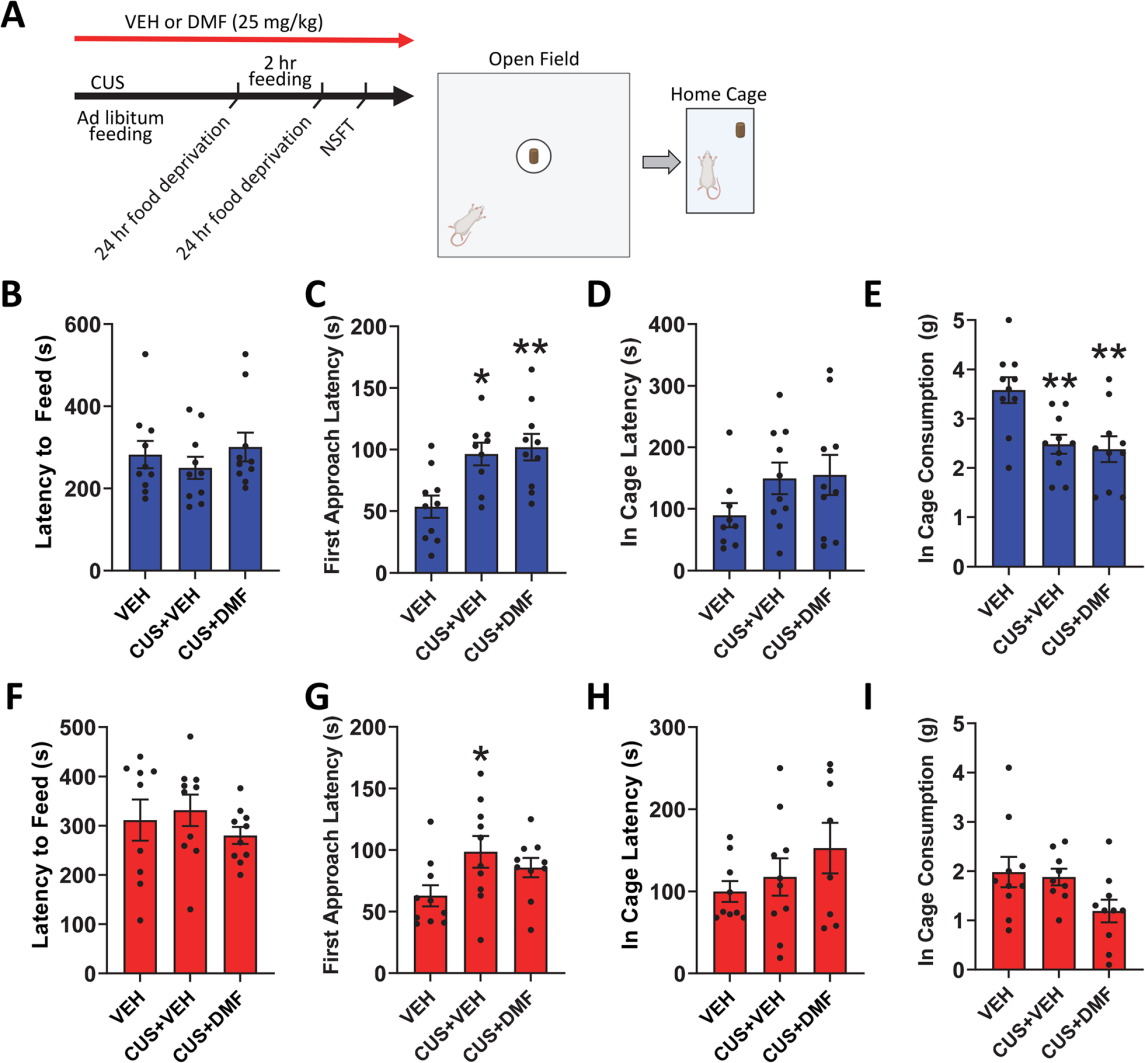
The effect of DMF treatment on CUS-induced anxiety-like behaviours in the novelty suppressed feeding task (NSFT). **A** Task timeline and graphics. **B** In male rats, there were no group differences in the latency to feed in the lightened open field arena in the NSFT. **C** However, the latency to first approach the centre of the arena was increased with CUS and unchanged with DMF treatment. **D** Male in-cage feeding latency showed no group differences after the NSFT, **E** but CUS reduced in-cage food consumption, an effect unaltered by DMF treatment. **F** During the NSFT, the latency to feed in the lightened open field arena showed no group differences in female rats. **G** Whilst CUS increased the latency to first approach the centre of the arena, there was no effect of DMF. After the NSFT, there was no group differences in the female **H** in-cage feeding latency or **I** in-cage food consumption. The data are presented as mean ± SEM of N = 9–10 animals per group. *p < 0.05 and **p < 0.01 compared to vehicle. A one-way ANOVA followed by a Tukey or Games-Howell post-hoc test was used for all analyses

The ability of DMF to ameliorate CUS-induced anxiety-like behaviour was next evaluated in the novelty suppressed feeding task and in-cage feeding (Fig. 2A). In male rats, the latency to feed in the lightened open field arena did not differ between groups (Fig. 2B). However, CUS did increase the latency to approach the centre of the arena (p = 0.01), an effect unaltered with DMF treatment [F(2.26) = 7.5, *p* = 0.003, Fig. 2C]. Following the task, male in-cage feeding latency showed no group differences (Fig. 2D), however in-cage food consumption was suppressed by CUS (*p* = 0.009) with no effect of DMF (F(2, 27) = 7.1, *p* = 0.002; Fig. 2E). In female rats (Fig. 2F–I), no group differences for any of the measures were evident except for latency to approach in the novelty suppressed feeding task whereby CUS increased the centre approach time (*p* = 0.04, Fig. 2G).

**Fig. 3:**
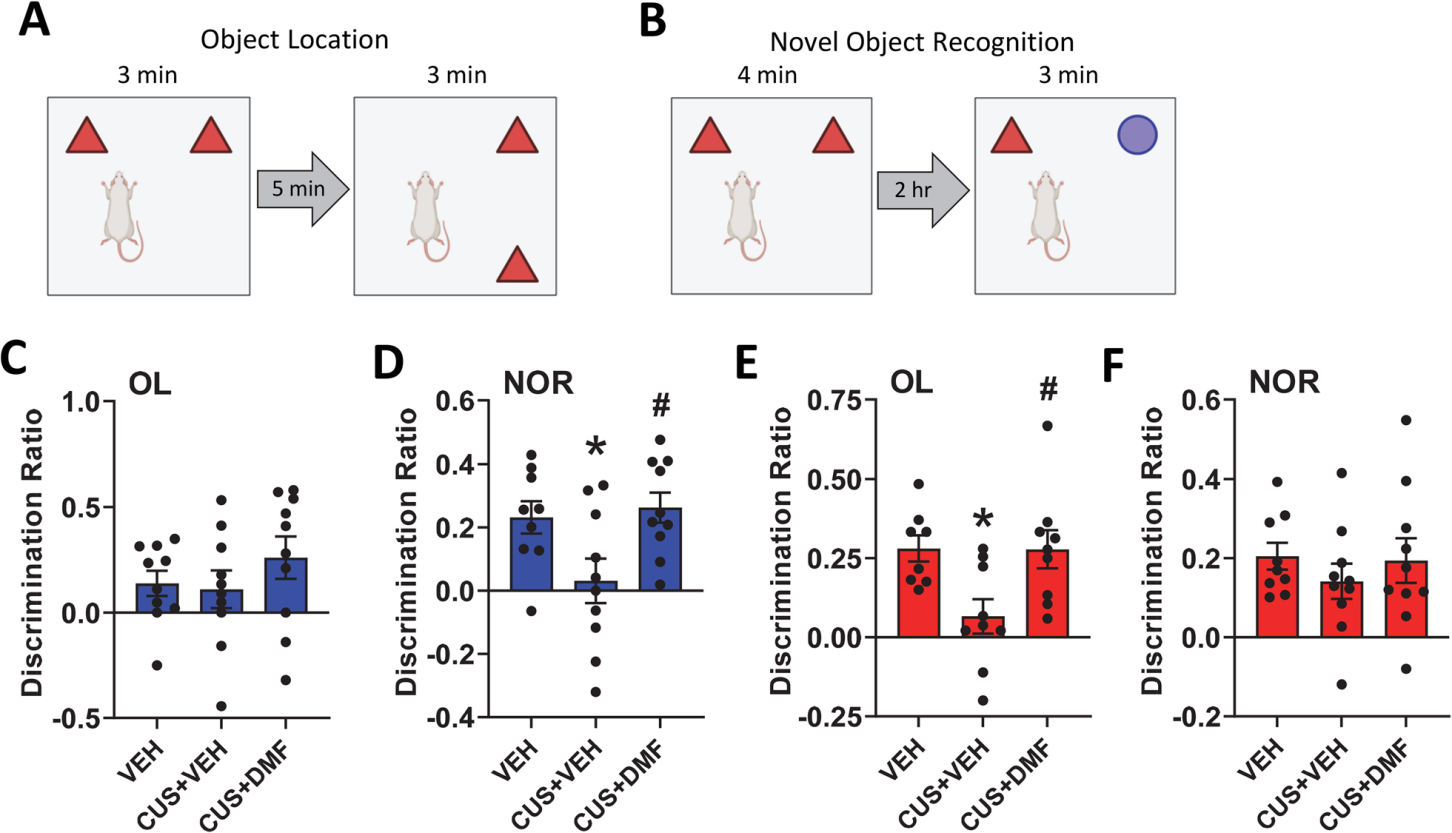
The effect of DMF treatment on CUS-induced learning and memory deficits. **A**, **B** Graphics for the object location and novel object recognition tests are shown. **C** Male animals displayed no group differences in the object location test. **D** In male rats, CUS induced deficits in the novel object recognition test that were rescued by DMF treatment. **E** In female rats, deficits in the object location test were observed with CUS exposure and these were alleviated by DMF treatment. **F** Female rats displayed no group differences in the novel object recognition test. The data are presented as mean ± SEM of N = 9–10 animals per group. *p < 0.05 compared to vehicle and #p < 0.05 compared to CUS + vehicle. A one-way ANOVA followed by a Tukey or Games-Howell post-hoc test was used for all analyses

To assess spatial or recognition learning and memory the object location and novel object recognition tests were used, respectively (Fig. 3A, B). CUS with or without DMF had no significant effect on spatial learning and memory in male rats (Fig. 3C). In the novel object recognition test, CUS induced deficits in recognition learning and memory (*p* = 0.05) that were normalized by DMF (F(2,24) = 4.8, *p* = 0.02, Fig. 3D). Conversely, in the female animals, CUS exposure resulted in object location memory deficits (*p* = 0.02) that were alleviated by DMF (F(2, 25) = 6.0, *p* = 0.007, Fig. 3E), with no group effects in the novel object recognition test (Fig. 3F). Collectively, these behavioural results indicate that DMF could ameliorate CUS-induced depression-like behaviour in male, but not female, rats. Further, in both sexes DMF had no impact on CUS-induced anxiety-like behaviour, whilst improvements in CUS-induced learning and memory deficits were evident.

**Fig. 4:**
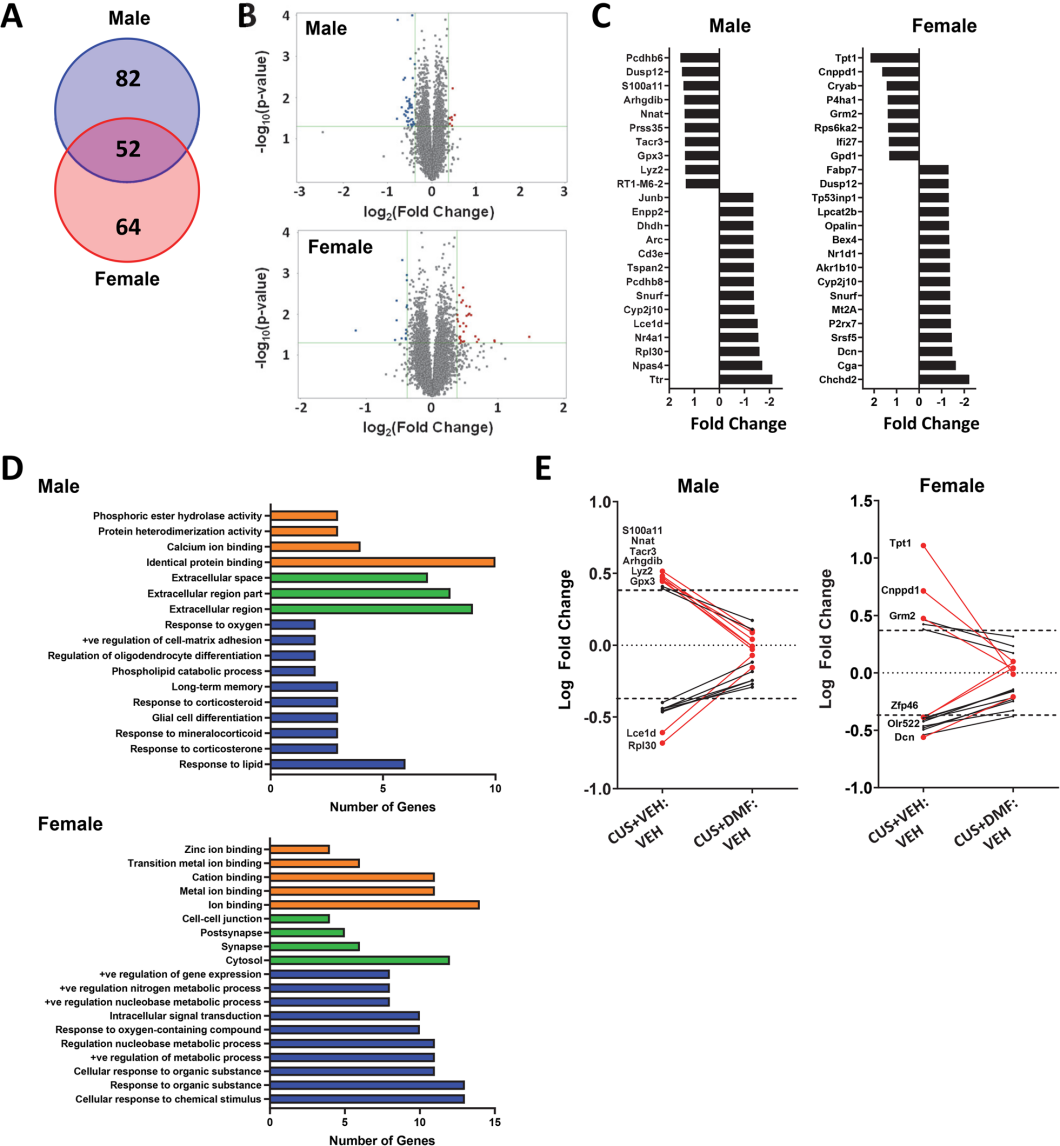
Sex-specific alterations in hippocampal gene expression in response to CUS and the mitigating effects of DMF. **A** The Venn diagram depicts the number of transcripts with a log_2_ fold change ≥ 0.4 or ≤ − 0.4 in males (blue) and females (red) following CUS. **B** Volcano plots represent the -log_10_(p-value) against the log_2_(fold change) magnitude of transcripts altered by CUS in males (top panel) and females (bottom panel). **C** The top 24 differentially expressed genes for each sex following CUS. **D** Gene Ontology analysis identified significantly enriched terms from males (top panel) and females (bottom panel) in the categories of molecular function (orange), cellular components (green), and biological processes (blue). **E** DMF normalization of CUS-induced changes in gene expression, focusing on transcripts with a minimal log_2_ fold correction of ≥ 0.3 or ≤ − 0.3

Gene expression changes in response to CUS and the impacts of DMF were assessed in HIP tissue. There were 134 genes altered in the HIP of CUS-exposed male rats and 116 genes altered in CUS-exposed female rats with a log_2_ fold change ≥ 0.4 or ≤ −0.4 (p < 0.05) (Fig. 4A). Overall, CUS induced a downregulation in male HIP and an upregulation in female HIP of the majority of gene transcripts (Fig. 4B). Of the genes altered by CUS, 52 transcripts were changed in both sexes (Fig. 4A) with the majority identified as unknown. The 6 known gene transcripts and their encoded protein function are described in Table 1. The gene *cnppd1* was upregulated in male and female HIP tissue by CUS and the genes *chchd2*, *arhgdib*, *opalin*, *cyp2j10* were downregulated in both sexes. The gene *dusp12* was regulated in a sex-dependent manner, with elevated HIP expression in males and lower expression in females.

**Table 1:** Gene transcripts altered in both male and female rats following CUS

Male	Female	Gene	Encoded protein function
↑	↑	*Cnppd1*	Cyclin Pas1/PHO80 domain containing 1; function in brain unknown; predicted to be involved in the regulation of cyclin dependent kinase activity
↑	↓	*Dusp12*	Dual specificity phosphatase 12; MAPK phosphatase; protective against endothelial cell inflammation and oxidative stress
↓	↓	*Chchd2*	Coiled-Coil-Helix-Coiled-Coil-Helix domain containing 2; mediator of oxidative phosphorylation and repressor of the mitochondrial stress response; implicated in Parkinson’s disease
↑	↓	*Arhgdib*	Rho GDP dissociation inhibitor beta; involved in immune and inflammatory responses
↓	↓	*Opalin*	Oligodendrocytic myelin paranodal and inner loop protein; transmembrane sialylglycoprotein; enriched in oligodendrocytes and involved in myelin stabilization
↓	↓	*Cyp2J10*	Cytochrome p450 enzyme; implicated in antidepressant responses

The top 24 most differentially expressed genes following CUS for each sex are depicted in Fig. 4C. GO analysis revealed enriched biological processes implicated in CUS-induced changes in male HIP that included processes such as “long-term memory”, “corticosteroid responses”, “glial cell differentiation”, and “oligodendrocyte differentiation” (Fig. 4D). In female HIP, the enriched biological processes were distinct and included “response to metabolic processes”, “response to organic substance”, and “response to chemical stimulus”. To evaluate the impact of DMF on CUS-mediated gene expression changes, normalization of these CUS-induced alterations was examined, with transcripts showing a minimal log_2_ fold correction of ≥ 0.3 or ≤ − 0.3 shown (Fig. 4E). In male HIP, 8 gene transcripts altered by CUS were normalized by DMF with most of these transcripts, including *nnat*, *arhgdib*, *lyz2*, *gpx3*, and *s100a11*, involved in immune or inflammatory responses or in oxidative stress (Table 2).

**Table 2:** Gene transcripts normalized by DMF in CUS-exposed male rats (absolute ΔLogFC > 0.3)

GENE	CUS RESPONSE	Encoded protein function
*Nnat*	↑	Neuronatin; ER membrane protein and linked to ER stress; involved in inflammation, calcium signalling, and glucose signalling
*Tacr3*	↑	Tachykinin/neurokinin 3 receptor; promotes acetylcholine release; involved in learning and memory, implicated in anxiety and depression
*Arhgdib*	↑	Rho GDP dissociation inhibitor beta; involved in immune and inflammatory responses
*Lyz2*	↑	Lysozyme 2; marker of microglial activation; linked to immune and inflammatory responses
*Gpx3*	↑	Glutathione peroxidase 3; known target gene of Nrf2; selenoprotein of the glutathione peroxidase family; antioxidant enzyme
*S100a11*	↑	Calgizarrin; calcium binding protein; implicated in pro-inflammatory processes
*Rpl30*	↓	Ribosomal protein L30; component of 60S subunit
*Lce1d*	↓	Late cornified envelope 1d; known target gene of Nrf2 in keratinocytes; function in brain unknown

DMF-induced normalization of CUS-mediated upregulation of *tacr3*, which encodes the tachykinin (neurokinin 3) receptor implicated in learning and memory, as well as depression and anxiety, was also evident. The genes normalized by DMF in female rat HIP were distinct from the males (Table 3) with three of these, *tpt1*, *dcn*, and *grm2* appearing to play a role in inflammatory/immune or antioxidant responses. *Grm2*, which encodes for the metabotropic glutamate receptor 2, has also been implicated in depression and anxiety. Together these findings indicate that DMF normalizes some of the gene expression changes in HIP induced by CUS, although these changes were distinct in male and female rats.

**Table 3:** Gene transcripts normalized by DMF in CUS-exposed female rats (absolute ΔLogFC > 0.3)

GENE	CUS RESPONSE	Encoded protein function
*Tpt1*	↑	Translationally-controlled tumor protein 1; Diverse functions including DNA damage repair, autophagy, protein degradation; antioxidant properties and implicated in immune responses
*Cnppd1*	↑	Cyclin Pas1/PHO80 domain containing 1; function in brain unknown; predicted to be involved in the regulation of cyclin dependent kinase activity
*Grm2*	↑	Metabotropic glutamate receptor 2; activation is anti-inflammatory; implicated in depression, stress resilience and vulnerability, anxiety
*Zfp46*	↓	Zinc finger protein 46, transcription factor; biological functional pathways unknown
*Olr522*	↓	Encodes for an olfactory receptor; specific function unknown although several functions for non-chemosensory regions in brain have been characterized
*Dcn*	↓	Decorin; extracellular matrix proteoglycan; plays a key role in inflammatory and autoimmune disorders

## Discussion

In the present study, we demonstrated that DMF treatment during CUS exposure had antidepressant effects in male rats only with no anxiolytic effects in either sex. CUS impaired recognition and spatial learning and memory in male and female rats, respectively; deficits that were rescued with DMF treatment. Further, sex-specific alterations in HIP gene expression following CUS exposure were identified, several of which were normalized in animals treated with DMF. Notably, the majority of the differentially expressed genes in the male rats were related to inflammatory or immune responses. Collectively, these findings suggest that the role of immune processes in depression is sexually dimorphic, with a potentially greater role in males than in females.

Evidence supports an association between immune system dysfunction and depression [14]. As such the sex-specific effects of DMF, a potent Nrf2 activator that promotes antioxidant and anti-inflammatory mechanisms, on CUS-induced behavioural responses were explored. Here, we report that male and female rats exhibited despair- and anhedonia-like behaviours following CUS, supporting findings from previous investigations of sex differences in animal models of stress and depression-like behaviours [49, 56–61]. Regarding anhedonia-like behaviour, as measured by the sucrose preference test, it should be noted that the growth of the CUS-exposed male rats was also lower and thus may relate to the total amount of sucrose consumed. However, this consideration would not apply to females given the lack of difference in body weight across treatment groups.

DMF treatment prevented the development of depression-like behaviours in male, but not female, rats. The antidepressant effects of DMF treatment in CUS-exposed male rats are in line with previous reports [23, 41, 42]; however to our knowledge, this is the first study to report a lack of antidepressant efficacy in CUS-exposed female rats. Other reports have shown limited efficacy of DMF treatment in female rodents in other models of neuroinflammation, including the EAE rat model [43] and a transgenic mouse model of AD [44].

The development of depression-like behaviours has been linked to HIP microglial activation as it results in the release of inflammatory factors that contribute to impaired neuroplasticity and cognition [62, 63]. In addition, anti-inflammatory drugs have been shown to attenuate depressive symptoms mediated by microglial activation [64, 65]. In support of the idea that stress-induced proinflammatory mechanisms play a greater role in depression-like behaviours in male rats, our gene expression analysis revealed that most CUS-altered transcripts, normalized with DMF treatment in males, were associated with inflammatory responses or in oxidative stress. One such gene transcript was *arhgdib,* which was upregulated in stressed males and was normalized by DMF treatment in males. *Arhgdib,* which encodes Rho GDP-dissociation inhibitor β (Rho-GDI2), has been associated with oxidative stress and inflammatory responses mediated by microglia in the entorhinal, frontal, and temporal cortices of human AD patients [66]. Elevated Rho GDI2 protein expression in HIP microglia was observed in male indoleamine-pyrrole 2,3 dioxygenase (IDO1)-knockout mice, a model of inflammation-associated depression [67]. Conversely, female rats exhibited a CUS-induced downregulation in *arhgdib* gene expression, which may suggest a reduced or lack of an inflammatory state in females exhibiting a depressive phenotype. In support of this, transgenic inactivation of astrocyte nuclear factor kappa-light-chain-enhancer of activated B cells (NF-kB) in an EAE mouse model resulted in a concomitant reduction in CNS inflammation and *arhgdib* gene expression [68].

In males, the *nnat* gene transcript emerged as a potential contributor to depression-like behaviours, with CUS inducing a male-specific upregulation in *nnat* expression, subsequently rescued by DMF treatment. *Nnat* encodes neuronatin, an endoplasmic reticulum (ER) membrane protein highly expressed in the brain, implicated in cell migration, neural induction of stem cells, and maintenance of synaptic plasticity [69, 70]. Human patients with MDD exhibit increased methylation of the *nnat* promoter, indicative of repressed gene transcription, with a significant association between elevated methylation and reduced self-reported depressive symptoms following electroconvulsive therapy [71]. Further, in a stressful social loss rat model of depression, socially dominant and subordinate (depressed) male animals had lower and higher levels of the *nnat* transcript, respectively, within the posterior cortex [72]. This is consistent with our findings, suggesting that upregulated *nnat* expression is associated with a pro-depressive phenotype. Given that DMF treatment normalized *nnat* transcripts and depression-like behaviours, it is likely that proinflammatory mechanisms link this gene transcript with depression. Indeed, neuronatin activates NF-κB in endothelial cells, resulting in increased inflammatory cytokine gene expression [73] and its expression has been associated with increased inflammatory responses in adipose tissue [74]. In addition, binding sites on the *nnat* promoter have been confirmed for the transcription factors Jun and Stat3, known to promote inflammatory signalling [74]. Interestingly, within the rat HIP, *nnat* mRNA expression was restricted to the CA2 and CA3 regions [69].

In CUS-exposed male rats, DMF normalized gene trancripts such as *s100a11* and *lyz2*, which may contribute to depression-like behaviours. The *s100a11* gene encodes calgizarrin, a calcium-binding protein that involved in inflammatory responses [75]. S100a11, induced by inflammatory cytokines, modulates p38 signalling to enhance inflammatory gene expression [75]. Moreover, in glioblastoma cells, s100a11 was shown to activate NF-κB via annexin-2, and NF-κB in turn had a positive feedback effect to increase s100a11 expression [76]. Although s100a11 has not been previously linked to depression, s100a11 is upregulated in neurological and inflammatory diseases, including glioblastomas [76], amyotrophic lateral sclerosis [77], rheumatoid arthritis [78], and autoimmune encephalitis [79]. In addition, its relative protein, s100a10 (p11), is widely implicated in depression-like behaviours in a region-specific manner [80, 81]. *Lyz2* encodes for Lysozyme M (LysM), an innate immune system enzyme and microglia activation marker [82]. Recently, Li et al. [83] reported that CUS-exposed male mice exhibiting depression-like behaviours had elevated *lyz2* mRNA expression in the HIP, which parallels our findings. DMF also normalized gene transcripts in CUS-exposed female rats; however, as DMF did not elicit antidepressant effects in females, these genes may not underlie depression-like behaviours in this model.

CUS induced anxiety-like behaviours in both sexes. While this finding matches several previous studies [58, 84–86], conflicting evidence of sex-specific [56, 60] and no anxiety-like behaviours [49] following stress paradigms have also been reported. Notably, anxiety-like behaviours in either sex were not rescued with DMF treatment; however, therapeutic effects on learning and memory deficits were evident. Clinical [87, 88] and preclinical [89–91] studies support that learning and memory are impaired in depression. Moreover, DMF treatment reportedly improved learning and memory deficits in other disease models, including AD [45, 92, 93], EAE [94], systemic immune challenge [95], and hypothyroidism [96]. In the present study, only male rats were found to have deficits in recognition learning and memory following CUS; deficits that were not present in DMF-treated males exposed to CUS. This finding agrees with those from de Souza et al. [42] whereby DMF reversed CUS-induced memory alterations in the novel object recognition test in male mice. Further, these sex differences in CUS-induced recognition learning and memory deficits mostly parallel previous findings in the literature [97–101], although conflicting evidence does exist [102]. With regard to spatial learning and memory, deficits were evident in CUS-exposed female rats only and these were rescued with DMF treatment. This contradicts the current understanding of stress-induced enhancement of spatial learning and memory in female rodents [99, 100, 103–105]. Notably, these previous studies used different stress-based protocols of varying amounts and durations of stress, which has been shown to differentially alter learning and memory in both sexes [103]. For example, one week, two weeks, and three weeks of restraint stress had no impact, enhanced, and impaired spatial learning and memory, respectively, in male rats [106]. Similarly, 6 h, but not 2 h, of intermittent restraint stress impaired spatial learning and memory in male rats [105]. Further, 6 weeks of intermittent restraint stress had no impact on spatial learning and memory in either sex, whilst 3 weeks of unpredictable intermittent stressors induced spatial learning and memory deficits in male, but not female, rats [105]. Collectively, this suggests that the discrepancy in findings may be a product of the implemented stress protocol.

The observed findings regarding learning and memory may be partially explained through concurrent alterations in gene expression. In males, elevated *nnat* expression has been implicated in recognition learning and memory. Specifically, increased *nnat* mRNA expression and protein abundance in the HIP and cortex have been shown in both apolipoprotein-D KO mice [107] and a transgenic mouse model of AD [108], both exhibiting deficits in recognition learning and memory tests. In addition, knocking down the level of *nnat* in transgenic AD mice rescued recognition learning and memory deficits [108]. *Tacr3,* another gene upregulated by CUS in males and subsequently normalized with DMF, has been implicated in learning and memory. Agonism of the tachykinin/neurokinin 3 receptor (NK3R), encoded by *tacr3*, has consistently been reported to increase cholinergic neurotransmission in the HIP, frontal cortex, and amygdala [109–111], thereby ameliorating age-related [109, 110] and scopolamine-induced [112] deficits in recognition and spatial learning and memory. Of note, pharmacological NK3R agonism in aged rats reduces *tacr3* mRNA expression in the HIP [110]. Therefore, the upregulation in HIP *tacr3* gene expression may be related to the recognition learning and memory deficits observed in this study. Although current evidence supports a role for NK3R in both recognition and spatial learning and memory in male rats [109, 110, 112, 113], this mechanism has not been investigated in the context of stress and depression. Thus, it is possible that NK3R signalling mediates task-specific learning and memory in a different pathological state.

In female rats exposed to CUS, upregulation of *grm2* expression may have contributed to the observed deficits in spatial learning and memory, given the established role of the glutamatergic system in learning and memory [114]. *Grm2* encodes for the metabotropic glutamate receptor 2 (mGlu2R), a group II mGluR that negatively regulates adenylate cyclase, leading to a reduction in intracellular cyclic AMP levels [114]. Group II receptors, predominantly located on presynaptic terminals in the HIP, PFC, and amygdala [115], modulate glutamatergic neurotransmission, long-term potentiation, and memory consolidation [114–116]. In line with this, agonism of group II mGluR (mGluR2/3) impaired spatial learning and memory in mice, whilst antagonism of mGluR2/3 [115, 117] and the knockout of mGluR2 [116, 118] improved and had no effect on this measure, respectively. Despite studies using drugs that affect both group II mGluR, the mGluR2 is predominantly expressed in HIP pyramidal neurons and is likely the receptor subtype mediating these effects [119]. Thus, it follows that increased *grm2* expression in the HIP of females may contribute to the spatial learning and memory deficits exhibited.

Last, it may be of note that sex hormones have been found to influence microglia morphology and function [120], and thus are an important consideration when assessing sex differences in immune responses. However, in the context of female stress responses, results from our previous work [49] showed that CUS significantly increased estrous cycle length with some rats showing a total loss of cycling, effects that began early in the CUS paradigm. Further, we showed that depression- and anxiety-like behaviours were not significantly different across animals in the different stages of the estrous cycle. As such, it is unlikely that the female-specific results described here were impacted by estrous cycling.

### Perspectives and significance

With the high rates of treatment resistance and relapse associated with current antidepressant pharmacotherapies [10, 11] and the greater prevalence of depression in woman compared to men [8], the need for novel effective sex-specific antidepressant treatments is substantial. This is the first study to investigate the sex-specific differences in the antidepressant potential of DMF using the CUS rodent model of depression. Our findings support the notion that DMF treatment has male-specific antidepressant properties and can also rescue sex-specific deficits in learning and memory. Given that DMF is a currently approved therapeutic for the treatment of relapse-remitting multiple sclerosis (RRMS) and has been associated with improvements in self-reported depression symptoms and quality of life in RRMS patients [121], it stands that DMF treatment could serve as a practical monotherapy or adjunct therapy for male patients with MDD, and as an adjunct therapy in women experiencing cognitive deficits. Furthermore, our transcriptomic results highlight the sexual dimorphisms of the neurobiological underpinnings of depression, emphasizing the ongoing necessity for studies focused on better understanding the role of sex in the pathophysiology of neuropsychiatric disorders.

## Data Availability

The datasets used and/or analyzed during the current study are available at the OSF repository osf.io/s49fe with gene expression data also available on the Gene Expression Omnibus.
