# Correction: Nrf2 activation rescues stress-induced depression-like behaviour and inflammatory responses in male but not female rats

**DOI:** 10.1186/s13293-024-00605-3

**Published:** 2024-04-03

**Authors:** Ryan T. McCallum, Rachel-Karson Thériault, Joshua D. Manduca, Isaac S. B. Russell, Angel M. Culmer, Janan Shoja Doost, Tami A. Martino, Melissa L. Perreault

**Affiliations:** Department of Biomedical Sciences, University of Guelph, 50 Stone Rd. E, Guelph, ON N1G 2W1 Canada

**Correction: Biol Sex Differ 15, 16 (2024)**

10.1186/s13293-024-00589-0

Following publication of the original article [1], the authors reported an error in the methodology for calculating the discrimination ratio in the novel object recognition and object location tests.

**Incorrect**: The discrimination ratio was calculated as the time of exploration of relocated object/total object exploration time*100.

**Correct**: For the novel object recognition test the discrimination ratio was calculated as the time exploring the novel object – the time exploring the familiar object/total exploration time.

For the object location test the discrimination ratio was calculated as the time exploring the novel location – the time exploring the familiar location/total exploration time.

The authors also report an error in sampling for original Figs. 4 and 5. Therefore, all of the original microglia data in the results and figures have been removed and Fig. 6 renumbered as Fig. 4. The associated immunohistochemistry methodologies were removed, and any associated discussion of the microglia findings deleted. References were renumbered and the Abstract and Highlights also revised to remove the microglia findings.
